# Unveiling the hidden struggles: A cross-sectional study on the mental health crisis among Chinese medical residents

**DOI:** 10.1097/MD.0000000000045500

**Published:** 2025-11-14

**Authors:** Pan Su, Xiaoyan Zhu, Xuan Zheng, Neng Liu, Rong Li, Aimin Wang

**Affiliations:** a Teaching and Research Section of Clinical Nursing, Xiangya Hospital, Central South University, Changsha, China; b Graduate Department, Xiangya Hospital, Central South University, Changsha, China; c Resident Standardized Training Office, Hunan University of Medicine General Hospital, Changsha, China; d Post-graduation Education Office, Xiangya Hospital, Central South University, Changsha, China; e Emergency Department, Xiangya Hospital, Central South University, Changsha, China.

**Keywords:** anxiety, depression, medical residents, mental health

## Abstract

Standardized residency training is a crucial component of medical education. However, residents commonly face heavy clinical workloads, occupational stress, and insufficient social support, contributing to a high prevalence of negative emotions such as depression, anxiety, and stress. These psychological issues significantly threaten personal well-being and the quality of medical care. International studies have documented prominent mental health challenges among medical residents; yet, empirical research within the Chinese context remains limited, particularly regarding the mechanisms involving social support, workload, and institutional design. This study aimed to evaluate the prevalence of negative emotions among residents undergoing standardized residency training, identify key influencing factors, and to provide a basis for developing targeted intervention strategies in the future. A cross-sectional study was conducted involving 514 residents from 3 general hospitals in Hunan Province. Data were collected using a general information questionnaire, the Depression Anxiety Stress Scales-21, and a residency training-specific questionnaire. Data analysis was performed using SPSS version 26.0 to assess the residents’ mental health status and identify associated influencing factors. The prevalence rates for depression, anxiety, and stress among residents were 86.4%, 92.2%, and 90.7%, respectively. Moderate-to-severe cases accounted for 19.8% (depression), 29.6% (anxiety), and 13.0% (stress). Factors significantly associated with negative emotions included interpersonal stress (odds ratios [OR] = 2.20–2.87), emotional stress (OR = 3.89), frequent extra shifts (OR = 4.85–5.42), duration of training (OR = 1.36), and low satisfaction with residency training (OR = 2.17–3.11). Conversely, positive perceptions regarding the value of residency training were protective factors (OR = 0.21–0.54). Negative emotions are prevalent among medical residents and influenced by multiple factors. Our findings indicate that interventions should prioritize mitigating interpersonal and emotional stressors, reducing non-rostered workloads, and fundamentally enhancing trainees’ satisfaction and sense of value within the training program to safeguard their mental well-being and, by extension, patient care quality.

## 1. Introduction

Standardized residency training is a critical component of the medical education system, designed to enhance residents’ clinical practice skills and professional competencies through structured training programs. During the 3-year training period, residents must complete 33 months of competency-oriented standardized residency training while concurrently fulfilling clinical duties assigned by hospitals. However, heavy academic workloads, complex clinical environments, and career development pressures pose significant mental health challenges for residents. International studies have consistently demonstrated that burnout, anxiety, and depression among medical residents are considerably higher compared to the general population, highlighting a critical issue in medical education. For instance, a systematic review reported the prevalence of depression among Saudi Arabian residents to range from 28% to 70.6%, with similarly elevated anxiety and stress levels.^[1]^ In China, residents’ mental health issues also warrant attention. Previous research indicated a high turnover intention rate of 47.87% among Chinese residents undergoing standardized training, primarily driven by burnout and psychological distress.^[2]^

Currently, standardized residency training in China faces dual challenges: on the one hand, there is an increasing demand within the healthcare system for high-quality clinical professionals; on the other hand, residents’ psychological health problems are becoming increasingly prominent. Literature reveals that the burnout rates among Chinese residents are generally higher than international averages, with higher risk particularly observed among female residents, junior trainees, and surgical specialties.^[3]^ At present, Chinese residents commonly face unique stressors, such as high-intensity shift schedules, uncertainty about career progression, and insufficient social support.^[4]^ Furthermore, residents working in high-risk departments, experiencing doctor–patient conflicts, prolonged working hours, continuous exposure to patient deaths, and implicit curricula are more likely to experience psychological distress and self-denial.^[5–7]^ These stressors ultimately compromise their professional competence.^[8]^

Negative emotions experienced by medical residents not only threaten their personal health but also potentially compromise the quality of healthcare services and patient safety. For example, burnout and emotional exhaustion significantly increase the probability of medical errors.^[8]^ Additionally, a lack of professional identity and pressures from hidden curricula within the workplace further exacerbate psychological burdens.^[9]^ While a growing body of literature has begun to document the prevalence of mental health issues among Chinese residents, the specific mechanisms through which social support, workload, and institutional design interact to affect negative emotions have not been fully explored and urgently need clarification.

Given this context, the present study aims to investigate medical residents undergoing standardized training at a tertiary hospital in China. The objectives include assessing the prevalence of negative emotions, identifying critical influencing factors, and proposing targeted intervention strategies. This study therefore seeks to identify levers for intervention within the unique context of Chinese standardized training. By pinpointing specific, modifiable factors (such as the perceived value of training and the burden of extra shifts^[9,10]^) we aim to provide actionable evidence for training administrators to tailor support mechanisms effectively.

## 2. Materials and methods

### 2.1. Participants

A convenience sampling method was employed to recruit participants from January 2021 to December 2024. All residents undergoing standardized residency training at 3 general hospitals in Hunan Province during this period were invited to participate voluntarily through online survey links distributed via institutional channels. Inclusion criteria were voluntary participation with informed consent and the ability to independently complete online questionnaires. Exclusion criteria were residents who interrupted or withdrew from the residency training program, those with a history of severe physical illness or psychiatric disorders, and respondents who submitted incomplete or logically inconsistent questionnaires (e.g., discrepancies between reported age and training duration). Based on previous research,^[11]^ the sample size for this cross-sectional study was calculated using the formula: $n=\frac{Z^{2}\cdot p\left(1-p\right)}{d^{2}}$. With a confidence interval (CI) of 95% (*Z* = 1.96), an allowable error of 0.05 (*d* = 0.05), and an expected anxiety prevalence of 30% (*P* = .3), the minimum required sample size was 323. Accounting for an anticipated invalid response rate of 20%, the final target sample size was determined to be 420. Ultimately, a total of 543 questionnaires were distributed. After removing invalid responses, 514 were included for analysis, yielding a high valid response rate of 94.7%, which meets the statistical requirements.

Although stratification by hospital or specialty was not performed during recruitment, the final sample of 514 participants exceeded the target and included residents from various training grades, specialties, and institutions, enhancing the representativeness of the sample.

### 2.2. Methods

#### 2.2.1. General information questionnaire

The questionnaire included demographic and professional data such as gender, age, training grade, specialty in residency training, identity of participation, highest education level upon entering residency, and the status of obtaining medical practitioner qualifications.

#### 2.2.2. Depression Anxiety Stress Scale (DASS-21) Chinese Version^[12,13]^

The DASS-21 comprises 21 items divided into 3 dimensions: depression (e.g., “I felt that life was meaningless”), anxiety (e.g., “I felt my heart palpitating”), and stress (e.g., “I found it difficult to relax”), with 7 items per dimension. Each item is scored using a 4-point Likert scale (0 = “never”, 3 = “almost always”). The total score for each dimension is multiplied by 2 and classified into severity levels (depression: normal 0 to 9, mild 10 to 13, moderate 14 to 20, severe 21 to 27, extremely severe ≥ 28; anxiety: normal 0 to 7, mild 8 to 9, moderate 10 to 14, severe 15 to 19, extremely severe ≥ 20; stress: normal 0 to 14, mild 15 to 18, moderate 19 to 25, severe 26 to 33, extremely severe ≥ 34). The Chinese version of DASS-21 has an overall Cronbach α of 0.96, with subscale reliability coefficients of 0.90 for depression, 0.89 for anxiety, and 0.90 for stress. The scale’s validity was verified through exploratory factor analysis (cumulative variance contribution rate of 72.3%).

#### 2.2.3. Residency training-related questionnaire

The questionnaire addressed 3 main aspects: workload, including weekly working hours, frequency of night shifts, and additional duties such as covering shifts or managing extra patients; psychosocial factors, including satisfaction with doctor–patient relationships (5-point scale), recognition from teaching supervisors (yes/no), and emotional stressors (e.g., relationship or marital issues, 5-point scale); and perceptions of training, including the perceived significance of residency training (5-point scale) and overall training satisfaction (5-point scale). The selection of these components was informed by previous studies investigating sources of stress and burnout among medical trainees.^[14–18]^

#### 2.2.4. Data collection

Electronic questionnaires were distributed via the Wenjuanxing platform, accompanied by explanations of study objectives and informed consent forms. After removing invalid questionnaires, data were exported to Excel for coding and organization.

### 2.3. Statistical method

Data analysis was performed using IBM SPSS Statistics for Windows, Version 26.0 (IBM Corp., Armonk) with 2-sided tests, and statistical significance was set at *P* < .05. Categorical data were expressed as frequencies and percentages, while continuous data were described using means ± standard deviation ($\bar{x}\pm s$). Chi-square tests or Fisher exact tests were used for categorical variables, and independent-sample *t* tests or Mann–Whitney *U* tests were employed for continuous variables. Variables with a *P*-value <.1 in univariate analyses were included in a multivariate logistic regression model, using backward stepwise selection (likelihood ratio method). Depression, anxiety, and stress were treated as binary dependent variables (present/absent), and odds ratios (OR) with 95% confidence intervals (CI) were calculated.

### 2.4. Quality control

To ensure content validity and relevance, the preliminary questionnaire underwent a rigorous development process. It was first evaluated by a panel of 5 experts (including 2 clinical psychology professors and 3 senior residency training program directors) to assess content validity, clarity, and relevance to the Chinese residency context. Based on their feedback, several items were reworded for precision (e.g., specifying “weekly working hours” to include clinical duties and academic activities), and the response scales were standardized to 5-point Likert scales for consistency. Subsequently, a pilot test with 20 residents (not included in the final sample) confirmed the clarity and face validity of the instrument, prompting minor phrasing adjustments before full deployment. During data collection, IP address restrictions were implemented to prevent duplicate submissions, and logical data checks were conducted to ensure consistency, such as matching age and training duration (e.g., training duration ≤ 3 years for residents aged 25 years). During the data analysis phase, data entry was independently performed by 2 researchers, and outliers were systematically verified.

## 3. Results

### 3.1. General characteristics of participants

Among the 514 participants, 240 (46.70%) were male and 274 (53.30%) were female. The largest age group was 23 to 26 years, comprising 394 participants (76.70%), followed by 27 to 30 years with 102 participants (19.80%). Most participants held a bachelor’s degree (286, 55.60%), followed by those enrolled in postgraduate studies (148, 28.80%). General practice was the most common training specialty (117, 22.76%), followed by internal medicine (112, 21.79%). Other specialties include anesthesiology, surgery, pediatrics, etc. The majority (333, 64.80%) were resident physicians who had obtained their practicing license. Second-year residents constituted the largest group (232, 45.10%), followed by first-year residents (155, 30.20%). Professional master’s students were the most common type of trainee (202, 39.30%), followed by socially recruited trainees (190, 37.00%). Results are presented in Table 1.

**Table 1:** General characteristics of participants.

No.	Item	Category	Count	Percentage
1	Gender	Male	240	46.70%
		Female	274	53.30%
2	Age	23–26	394	76.70%
		27–30	102	19.80%
		>30	18	3.50%
3	Education level	Bachelor’s degree	286	55.60%
		Current postgraduate student	148	28.80%
		Master’s degree	74	14.40%
		Doctoral degree	6	1.20%
4	Residency specialty	General practice	117	22.76%
		Internal medicine	112	21.79%
		Surgery	52	10.12%
		Pediatrics	47	9.14%
		Anesthesiology	52	10.12%
		Orthopedics	18	3.50%
		Obstetrics and gynecology	16	3.11%
		Emergency	16	3.11%
		Neurology	15	2.92%
		Radiology	14	2.72%
		Ophthalmology	12	2.33%
		Otorhinolaryngology	11	2.14%
		Ultrasound medicine	9	1.75%
		Critical care medicine	8	1.56%
		Psychiatry	7	1.36%
		Dermatology	4	0.78%
		Anesthesiology	4	0.78%
5	Medical practitioner qualification	Yes	333	64.80%
		No	181	35.20%
	Residency training year	1st year	155	30.20%
		2nd year	232	45.10%
		3rd year	127	24.70%
6	Training entry identity	Professional master’s student	202	39.30%
		Institution employee	11	2.10%
		Directed resident	111	21.60%
		Socially recruited trainee	190	37.00%

### 3.2. Mental health status

Among the 514 participants, the mean depression score was 7.30 ± 8.12 points, the mean anxiety score was 6.59 ± 7.67 points, and the mean stress score was 9.43 ± 9.02 points. According to the subscale classification criteria of the DASS-21, regarding depression levels among residents, 70 (13.6%) had normal levels, 342 (66.5%) were classified as mild, 69 (13.4%) as moderate, 10 (1.9%) as severe, and 23 (4.5%) as extremely severe. For anxiety, 40 (7.8%) had normal levels, 322 (62.6%) were mild, 93 (18.1%) moderate, 23 (4.5%) severe, and 36 (7.0%) extremely severe. In terms of stress, 48 (9.3%) showed normal levels, 399 (77.6%) were mild, 36 (7.0%) moderate, 18 (3.5%) severe, and 13 (2.5%) extremely severe. The results are presented in Table 2.

**Table 2:** DASS-21 scale scores.

Category	Depression	Anxiety	Stress
Normal	70 (13.6%)	40 (7.8%)	48 (9.3%)
Mild	342 (66.5%)	322 (62.6%)	399 (77.6%)
Moderate	69 (13.4%)	93 (18.1%)	36 (7.0%)
Severe	10 (1.9%)	23 (4.5%)	18 (3.5%)
Extremely severe	23 (4.5%)	36 (7.0%)	13 (2.5%)

### 3.3. Multivariate analysis of negative psychological emotions

Using negative psychological emotions as the dependent variable and incorporating the indicators with significant differences from univariate analysis as independent variables into the multivariate logistic regression model, the results revealed: “interpersonal stress: stressed doctor–patient relationships and not being recognized by patients and teachers” had a statistically significant impact on depression (OR = 2.204, 95% CI: 1.042–4.661, *P* < .05); “perception of residency training significance” was statistically associated with depression (*P* < .05); “satisfaction with residency training” demonstrated a significant effect on depression (OR = 2.780, 95% CI: 2.053–3.764, *P* < .0001). For stress, “covering shifts or managing patients for colleagues” showed a statistically significant association (OR = 5.574, 95% CI: 1.537–20.214, *P* < .05), and “satisfaction with residency training” also had a significant impact (OR = 3.243, 95% CI: 2.360–4.457, *P* < .05). Regarding anxiety, “residency year” was a statistically significant factor (OR = 1.356, 95% CI: 1.031–1.784, *P* < .05), as were “covering shifts or managing patients for colleagues” (OR = 4.846, 95% CI: 1.295–18.140, *P* < .05), “interpersonal stress: stressed doctor–patient relationships and not being recognized by patients and teachers” (OR = 2.869, 95% CI: 1.380–5.961, *P* < .05), “emotional stress” (OR = 3.892, 95% CI: 1.410–10.745, *P* < .05), and “perception of residency training significance” (*P* < .05). Additionally, “satisfaction with residency training” was significantly associated with anxiety (OR = 2.165, 95% CI: 1.639–2.859, *P* < .05). The results are presented in Table 3.

**Table 3:** Logistic regression analysis results.

Dependent variable	Selected independent variable	B	SE	Wald	*P*	OR	95% CI of OR
Depression	Residency year	0.28	0.147	3.622	.057	1.324	0.992–1.767
	Interpersonal stress	0.79	0.382	4.272	.039*	2.204	1.042–4.661
	Perception of residency training significance			14.616	.006*		
	Merely a compulsory learning process	-1.474	0.632	5.436	.02*	0.229	0.066–0.791
	Merely mechanical tasks assigned by supervisors	-0.618	0.636	0.943	.332	0.539	0.155–1.876
	Merely mechanical tasks assigned by supervisors	-1.386	0.723	3.675	.055	0.25	0.061–1.032
	Doctor’s responsibility to perform clinical work under supervision	-1.561	0.595	6.888	.009*	0.21	0.065–0.673
	Satisfaction with residency training	1.022	0.155	43.709	0*	2.78	2.053–3.764
Anxiety	Residency year	0.305	0.14	4.741	.029*	1.356	1.031–1.784
	Covering shifts or managing patients for colleagues	1.578	0.673	5.492	.019*	4.846	1.295–18.14
	Interpersonal stress: Stressed doctor–patient relationships and not being recognized by patients and teachers.	1.054	0.373	7.974	.005*	2.869	1.38–5.961
	Emotional stress	1.359	0.518	6.882	.009*	3.892	1.41–10.745
	Perception of residency training significance			11.13	.025*		
	Merely a compulsory learning process	-0.609	0.565	1.162	.281	0.544	0.18–1.646
	Merely mechanical tasks assigned by supervisors	-0.004	0.573	0	.994	0.996	0.324–3.058
	Still in student role, completing intern tasks	-0.676	0.663	1.04	.308	0.508	0.139–1.865
	Doctor’s responsibility to perform clinical work under supervision	-0.935	0.529	3.125	.077	0.393	0.139–1.107
	Satisfaction with residency training	0.772	0.142	29.624	0*	2.165	1.639–2.859
Stress	Weekly working hours	0.1	0.109	0.853	.356	1.106	0.893–1.368
	Research tasks	0.448	0.279	2.578	.108	1.566	0.906–2.707
	Covering shifts or managing patients for colleagues	1.69	0.656	6.646	.01*	5.421	1.51–9.596
	Interpersonal stress: stressed doctor–patient relationships and not being recognized by patients and teachers.	0.719	0.395	3.308	.069	2.052	0.946–4.453
	Satisfaction with residency training	1.134	0.167	45.821	0*	3.107	2.238–4.314

CI = confidence interval, DASS-21 = Depression Anxiety Stress Scales-21, OR = odds ratios.

* *P *< .05.

## 4. Discussion

### 4.1. Prevalence and characteristics of negative psychological emotions among residents

The prevalence rates of depression (86.4%), anxiety (92.2%), and stress (90.7%) observed in this study exceed those commonly reported in prior research among Chinese medical trainees.^[19,20]^ This discrepancy is likely not merely a function of assessment tools or timing, but rather a reflection of the unique composition of our cohort, which comprised a large proportion of individuals facing dual pressures from academic and clinical commitments.^[19,20]^ For instance, a meta-analysis by Zeng et al reported pooled prevalence rates of 29% for depression and 21% for anxiety among medical students in China,^[19]^ while studies focusing on residents during the COVID-19 pandemic also documented elevated but comparatively lower psychological symptoms.^[21,22]^ Our findings align with the recognized global prevalence of mental health issues among resident physicians. Studies in Saudi Arabia have reported depression prevalence rates ranging from 28% to 70.6% among residents.^[1]^ Cao Jie et al reported that 47.5% of residency trainees screened positive for mental health issues, and 63.9% exhibited high occupational stress, highlighting prominent psychological pressure and mental health concerns.^[23,24]^

The notably high prevalence of psychological issues among medical residents in this study may be attributed to several factors. First, the majority of participants in this study were either professional master’s students integrated into the standardized residency training or socially recruited trainees. These residents often experience a lack of belongingness and face dual pressures from academic studies and clinical responsibilities, leading to increased susceptibility to negative emotions. Zhu Xiaoyan et al demonstrated that under the “dual-track integration” training model, professional master’s students reported higher levels of depressive symptoms and perceived stress compared to social residency trainees.^[25]^ Additionally, graduate students at different stages of their training exhibit varying levels of stress and sources of stress.^[26]^ Second, general practice trainees constituted the largest proportion among residency specialties in this study. After graduation, general practitioners primarily serve at grassroots medical institutions, where they generally face lower compensation and uncertain career prospects, thereby intensifying their psychological burdens. Liu Fang et al found that 56.3% of general practice residents reported experiencing occupational burnout.^[27]^ Similarly, Xia Yu et al indicated that general practice residents have a relatively high rate of psychological issues, closely associated with personal factors and challenges encountered during residency training.^[11]^ Lastly, most residents surveyed were recruited from remote prefecture-level cities in western Hunan Province, characterized by relatively weaker foundational medical education. This background may contribute to greater psychological stress when residents encounter intensive clinical practices and complex clinical knowledge. For example, Xu Wei et al reported that 94.4% of residents nearing completion of their training exhibited anxiety, and 60.3% displayed depressive symptoms. Residents graduating from institutions with comparatively lower educational standards exhibited higher rates of anxiety and depression compared to those from higher-standard institutions.^[28]^

### 4.2. Analysis of factors influencing negative emotions among medical residents

Our study identified several factors contributing to negative emotions among medical residents, including high interpersonal stress (tense doctor–patient relationships and lack of recognition by patients and supervisors), significant emotional stress, low perceived meaning in daily residency training activities, frequent coverage of shifts or patient management tasks for colleagues, extended residency training duration, and overall low satisfaction with residency training. Interpersonal tensions and insufficient social support are significant sources of psychological stress for residents,^[29,30]^ while a lack of perceived value in clinical work and unclear career goals and training directions substantially decrease psychological health.^[31,32]^ Frequent additional shifts or managing extra patients may lead to extended working hours and overload, exacerbating physical and emotional exhaustion, thereby increasing stress and the risk of depression, insomnia, and occupational burnout.^[33,34]^ Notably, low overall satisfaction with residency training emerged as a common factor associated with depression, stress, and anxiety, reflecting deep-rooted structural deficiencies within the training system interacting with individual psychological capital. Studies indicate that residents who are dissatisfied with working or learning conditions and career choices report significantly higher levels of anxiety,^[30,35]^ likely because low satisfaction diminishes professional identity and self-efficacy, further intensifying psychological pressure. Therefore, management should carefully consider and systematically plan residents’ living and learning environments to enhance their overall satisfaction.

These findings indicate that the negative emotions experienced by residents result from multiple interacting factors. Not only are these emotions closely linked to external factors such as working environments and interpersonal relationships, but they are also influenced by internal cognitive factors and workload demands. To effectively alleviate this issue, it is recommended that management departments, hospitals, and health commissions collaboratively improve training systems and curriculum design, establishing a systematic mechanism for psychological health evaluation and intervention for residents. Specific measures should include optimizing doctor–patient communication, enhancing mentorship systems, rationalizing workload distribution, and building a comprehensive psychological support framework. Residency instructors and training mentors, as individuals in close daily contact with residents, should actively monitor their mental health, provide psychological education and emotional counseling, and offer support in academic, research, and social aspects to foster an inclusive, respectful, and innovative learning environment.^[36]^ Furthermore, residents themselves should continuously improve their professional skills and self-regulation capabilities to enhance self-efficacy and effectively manage psychological pressures encountered in both work and personal life.^[37]^

## 5. Conclusion

Our study highlights the widespread prevalence of depression, anxiety, and stress among medical residents undergoing standardized training, with incidence rates of 86.4%, 92.2%, and 90.7%, respectively, including significant proportions of moderate-to-severe cases. Key influencing factors identified include interpersonal stress, emotional stress, frequent additional shifts, prolonged training duration, low satisfaction with residency training, and perceptions regarding the significance of training. These findings underscore that residents’ mental health is affected by multiple internal and external factors, necessitating systematic interventions such as improving doctor–patient communication, enhancing mentorship support systems, rational workload distribution, and boosting overall training satisfaction. Future research may consider longitudinal tracking or interventional studies with expanded sample coverage to validate these findings’ generalizability. Our study provides critical evidence for improving residents’ psychological health, stabilizing the healthcare workforce, and enhancing service quality.

## Acknowledgments

The authors gratefully acknowledge all the medical residents who participated in this study. This work was supported by research grants from the China Medical Board and various teaching reform funds at the provincial and institutional levels. We also extend our appreciation to the residency training management offices of the participating hospitals for their valuable support and cooperation throughout this research.

## Author contributions

**Conceptualization:** Pan Su.

**Data curation:** Pan Su.

**Formal analysis:** Xiaoyan Zhu.

**Funding acquisition:** Xiaoyan Zhu.

**Investigation:** Xuan Zheng.

**Methodology:** Xuan Zheng.

**Validation:** Neng Liu, Rong Li, Aimin Wang.

**Visualization:** Neng Liu.

**Supervision:** Rong Li, Aimin Wang.
